# Fgf10 Signaling-Based Evidence for the Existence of an Embryonic Stage Distinct From the Pseudoglandular Stage During Mouse Lung Development

**DOI:** 10.3389/fcell.2020.576604

**Published:** 2020-10-22

**Authors:** Sara Taghizadeh, Matthew R. Jones, Ruth Olmer, Saskia Ulrich, Soula Danopoulos, Chengguo Shen, Chaolei Chen, Jochen Wilhelm, Ulrich Martin, Chengshui Chen, Denise Al Alam, Saverio Bellusci

**Affiliations:** ^1^Key laboratory of Interventional Pulmonology of Zhejiang Province, Department of Pulmonary and Critical Care Medicine, The First Affiliated Hospital of Wenzhou Medical University, Wenzhou, China; ^2^Cardio-Pulmonary Institute (CPI) and Department of Pulmonary and Critical Care Medicine and Infectious Diseases, Universities of Giessen and Marburg Lung Center (UGMLC), Member of the German Center for Lung Research (DZL), Justus Liebig University Giessen, Giessen, Germany; ^3^Leibniz Research Laboratories for Biotechnology and Artificial Organs (LEBAO), REBIRTH – Research Center for Translational and Regenerative Medicine, Biomedical Research in Endstage and Obstructive Lung Disease (BREATH), German Center for Lung Research (DZL), Department of Cardiothoracic, Transplantation and Vascular Surgery, Hannover Medical School, Hanover, Germany; ^4^Lundquist Institute for Biomedical Innovation at Harbor-UCLA Medical Center, Torrance, CA, United States

**Keywords:** FGF10, human lung, mouse lung, branching morphogenesis, embryonic phase

## Abstract

The existence during mouse lung development of an embryonic stage temporally and functionally distinct from the subsequent pseudoglandular stage has been proposed but never demonstrated; while studies in human embryonic lung tissue fail to recapitulate the molecular control of branching found in mice. Lung development in mice starts officially at embryonic day (E) 9.5 when on the ventral side of the primary foregut tube, both the trachea and the two primary lung buds emerge and elongate to form a completely separate structure from the foregut by E10. In the subsequent 6 days, the primary lung buds undergo an intense process of branching to form a ramified tree by E16.5. We used transgenic mice allowing to transiently inhibit endogenous fibroblast growth factor 10 (Fgf10) activity in mutant embryos at E9, E9.5, and E11 upon intraperitoneal exposure to doxycycline and examined the resulting lung phenotype at later developmental stages. We also determined using gene arrays the transcriptomic response of flow cytometry-isolated human alveolar epithelial progenitor cells derived from hESC or hiPSC, grown *in vitro* for 12 or 24 h, in the presence or absence of recombinant FGF10. Following injection at E9, the corresponding mutant lungs at E18.5 appear almost normal in size and shape but close up examination indicate failure of the right lung to undergo lobar septation. At E9.5, the lungs are slightly hypoplastic but display normal differentiation and functionality. However, at E11, the lungs show impaired branching and are no longer functional. Using gene array data, we report only a partial overlap between human and mouse in the genes previously shown to be regulated by Fgf10 at E12.5. This study supports the existence of an embryonic stage of lung development where Fgf10 signaling does not play a function in the branching process but rather in lobar septation. It also posits that functional comparisons between mouse and human organogenesis must account for these distinct stages.

## Introduction

Fibroblast growth factor 10 (Fgf10) is a secreted factor belonging to the Fgf family comprised of 22 different ligands and four receptors. Fgf10 secreted by mesenchymal cells acts on the adjacent epithelium in a paracrine manner through its receptor Fgfr2b to control the process of branching morphogenesis in different organs including the lung, pancreas, mammary gland, salivary and lacrimal glands during the early stages of mouse development. Genetic deletion in mouse of either *Fgf10* or its cognate receptor *Fgfr2b* result in agenesis of the aforementioned organs (Ornitz and Itoh, 2015).

Based on the conservation of the signaling pathways and biological processes governing organ development between human and mouse, the mouse model has been used extensively as a surrogate to study the role of Fgf signaling in organogenesis. For example, we have reported that Fgf10 deficiency in newborn mice leads to lethality upon hyperoxia exposure (Chao et al., 2015). Interestingly, decrease in FGF10 expression in the lungs is also observed in human patients displaying bronchopulmonary dysplasia (BPD) (Benjamin et al., 2007). BPD is characterized by arrested lung development at the so called “saccular stage” leading to a lung which has an “emphysematous-like” appearance. From a developmental point of view, this phenotype is caused by impaired formation of secondary septa which are transient structures leading to the subdivision of the sacs into smaller units called alveoli. Our mouse data suggest that Fgf10 is indeed critical for the alveologenesis phase to occur and has also a collateral impact on the formation of the vascular system (Chao et al., 2019). Therefore, while the role of Fgf10 appears to be consistent between human and mouse during alveologenesis, we recently reported that FGF10 displayed a discordant role in lung branching morphogenesis in human and mouse (Danopoulos et al., 2019). During lung development, the process of branching morphogenesis takes place during the so called “pseudoglandular stage” which in mice has been proposed to occur within a 7 days period from E9.5 through E16.5 while in humans, this stage lasts 13 weeks, from week 4 through week 17 after fertilization (Volckaert and De Langhe, 2015).

The expression of FGF10 and its receptors, FGFR1 and FGFR2, during human lung development are very similar to what is observed in mouse lungs, where *Fgf10* is highly expressed in the distal mesenchyme adjacent to epithelial buds (Bellusci et al., 1997). In addition, human *FGF10* expression is stable in the pseudoglandular stage and increases significantly in the canalicular stage (Al Alam et al., 2015). *FGF10* is detected from 10 weeks of gestation up to 21 weeks of gestation. Using human fetal lung explants cultured *in vitro*, we have reported that recombinant FGF10 is not sufficient to enhance the process of branching morphogenesis while it clearly triggers increased branching on E12.5 mouse lung (Danopoulos et al., 2019). This apparent difference in FGF10 activity between human and mouse could be explained by either an intrinsic and significant difference concerning the role of FGF10 between the two species during the corresponding pseudoglandular stages of lung development or, alternatively, that there is an additional phase of lung development in mice, and perhaps also in humans, called the “embryonic phase,” where Fgf10 is not regulating the process of branching *per se*. When this phase starts and ends in human and mouse is not clear. In addition, this phase is poorly defined both from a molecular and cellular point of view. We propose that the apparent discrepancy between the role of FGF10 in human and mouse could be reconciled by the existence of an embryonic phase where Fgf10 would not play a function in branching morphogenesis. To test this hypothesis, we used a genetically modified mouse line which allows the inducible, robust and ubiquitous expression of a soluble form of Fgfr2b which will inhibit Fgf10 ligand activity at the protein level. This line has been extensively used and validated in mouse to study the role of Fgfr2 signaling in multiple organs (Parsa et al., 2010; Danopoulos et al., 2013; Jones et al., 2019, 2020). In this study, we transiently inhibited endogenous Fgf10 activity in mutant embryos at E9, E9.5, and E11 following intra-peritoneal exposure of the embryos to doxycycline and examined the resulting lung phenotype at E18.5. We also determined, using gene arrays, the transcriptomic response in presence or absence of recombinant FGF10, after 12 or 24 h of culture *in vitro* of flow cytometry-isolated human alveolar epithelial progenitor cells derived from hESC or hiPSC. Our study supports the existence of an embryonic stage of lung development in mice where Fgf10 signaling does not play its previously described function in branching morphogenesis. Whether a comparable phase exists in humans is left to be determined.

## Results

### Transient Inhibition of Fgf10 Signaling at E9 Leads to Limb Agenesis but Normal Lung Development

**FIGURE 1:**
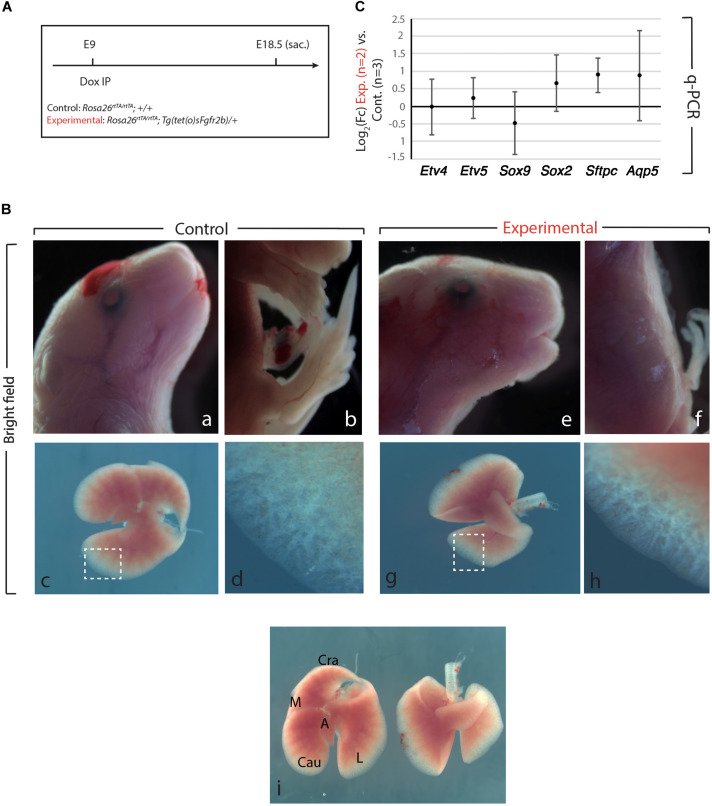
Fgfr2b signaling attenuation results in limb defects and defects in lobar septation. **(A)** Experimental plan for doxycycline injection at E9. Rosa26^*rtTA/rtTA*^; Tg (tet(O)sFgfr2b)/+ double transgenic system inducing, upon doxycycline exposure (via a single IP) in experimental group versus control group (Rosa26^*rtTA/rtTA*^; +/+) the expression of a soluble form of Fgfr2b acting as a dominant negative receptor. **(B)** Visibly different phenotypes in control group **(a–d)** compared to the experimental group **(e–h)**. Side-by-side comparison of control **(left)** and experimental lung **(i)**. Scale bar: **(a,b,e,f)** 2 mm; **(c,g,i)** 750 μm; **(d,h)** 150 μm. **(C)** Quantification of Fgf10 targets (*Etv4, Etv5, Sox2, Sox9, Sftpc*, and *Aqp5*) by qPCR normalized to *Hprt. n* = 2 in Experimental group and *n* = 3 in control group.

We generated Control (*Rosa26r^*tTA/rtTA*^; +/+*) and Experimental (*Rosa26^*rtTA/rtTA*^; Tg(sFgfr2b)/+*) mouse embryos by crossing *Rosa26^*rtTA/rtTA*^; Tg(sFgfr2b)/+* males with *Rosa26r^*tTA/rtTA*^; +/+* females. Pregnant females were then injected intraperitoneal (IP) at embryonic day (E) 9 with doxycycline (Figure 1A). In our experimental conditions, the expression of the soluble form of Fgfr2b peaks 6 h after Dox-IP injection and lasts for 24 h. We also reported that the inhibitory activity is reversible after this 24 h-time period (Danopoulos et al., 2013).

As the main Fgfr2b ligand expressed in the lung at this early developmental stage (E9.5–E12.5) is Fgf10 (Bellusci et al., 1997; Jones et al., 2019), our experimental approach leads to the inhibition of Fgf10 activity. We will therefore refer throughout this study to Fgf10 inhibition after doxycycline exposure. Figure 1B displays the phenotypic differences between experimental and control embryos. While the craniofacial features of experimental and control embryos are similar (Figures 1Ba,e), the limb agenesis phenotype in experimental embryos is striking (Figures 1Bb,f). Due to the importance of the role of Fgf10 signaling during the induction of the apical ectoderm ridge (AER) in the limb rudiment at E9.5 and E10 in the forelimb and hindlimb, respectively, the limb agenesis phenotype observed in the experimental embryos represents an excellent validation that the induced expression of soluble Fgfr2b is translated into a functional inhibition of Fgfr2b signaling. Interestingly, the experimental lungs were almost completely indistinguishable from control embryos both in size and shape at the exception of the absence of lobar septations allowing the separation of the caudal, medial and cranial lobes on the right lung (Figure 1Bi). qPCR analysis indicated no significant difference for the expression of *Etv4*, *Etv5*, *Sox9*, *Sox2*, *Sftpc*, and *Aqp5* [which are all target genes for Fgf10 signaling except for *Aqp5*, a gene encoding a marker of alveolar type 1 (AT1) cells] between Experimental and Control lungs (Figure 1C).

### Transient Inhibition of Fgf10 Signaling at E9.5 Leads to a Hypoplastic Lung but With Normal Epithelial Differentiation

**FIGURE 2:**
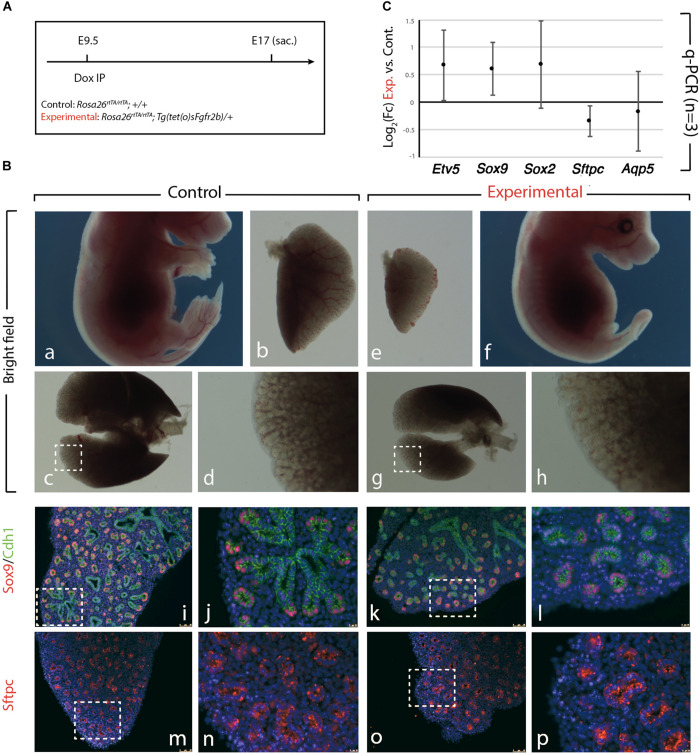
Inhibition of Fgfr2b signaling at E9.5 results in lobar defects but not branching defects. **(A)** IP injection at E9.5. **(B)** The effect of Fgfr2b blocking at E9.5, limb agenesis and defected branching morphogenesis in experimental group **(a–d)** vs. control group **(e–h)**. Characterization of epithelial cells in distal tip in two groups **(i–l)** and staining of the specific markers for distal progenitors and for AT2 cells, Sox9, and Sftpc, respectively **(m–p)**. Scale bar: **(a,f)** 5 mm; **(b,e)** 500 μm; **(c,g)** 750 μm; **(d,h)** 185 μm; **(i,k,m,o)** 150 μm; **(j,l,n,p)** 25 μm. **(C)** analysis of gene expression of collected lungs in two groups (*Etv5, Sox2, Sox9, Sftpc*, and *Aqp5*). *n* = 3 in both groups.

We further confirmed the results obtained upon Fgf10 inhibition at E9 (Figure 1), by injecting Dox-IP 12 h later, at E9.5 (Figure 2A) and examined the resulting phenotype at E17. Inhibition of Fgfr2b signaling following Dox-IP is expected to last 24 h, which in our experimental conditions corresponds to E10.5. This time period (E9.5–E10.5) overlaps with the timing of AER induction in the forelimb and hindlimb and as previously, we observe a limb agenesis phenotype (Figures 2Ba,f). Furthermore, macroscopic analysis of the experimental vs. control lungs indicates reduced lung size (Figures 2Bb,e,c,g), absence of accessory lobe and impaired lobar septation in the right lung (Figures 2Bc,g). Close up examination of the left lobe, which was reduced in size in the experimental vs. control lung, indicated no significant differences in the branching process (Figures 2Bd,h). Immunofluorescence analysis with Cdh1 (E-cadherin), and Sox9 antibodies to determine the status of the distal alveolar progenitors revealed no major differences between control and experimental lungs (Figures 2Bk,l). A similar result is obtained with Sftpc, an AT2 marker (Figures 2Bm–p). qRT-PCR analysis shows that similar to the E9 time point, the expression of *Etv5*, *Sox9*, *Sox2*, *Sftpc*, *and Aqp5* is not drastically affected between experimental and control lungs (Figure 2C). We conclude that during the E9–E10 period (Figure 1), the impact of Fgf10 signaling on the branching process is relatively minor. However, from E10 (Figure 2), Fgf10 signaling starts to be required for the branching process to occur. In addition, during the time period E9.5–E10.5, the effects of this inhibition are mostly reversible as indicated by normal epithelial differentiation.

### Transient Inhibition of Fgf10 Signaling at E11 Leads to an Arrest in Lung Development

**FIGURE 3:**
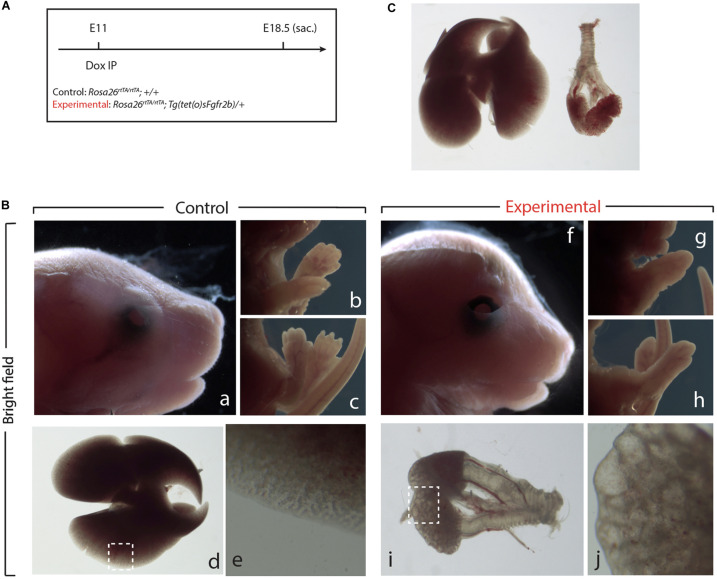
Inhibition of Fgfr2b signaling at E11 results in severe lungs defects. **(A)** Single Dox-IP injection at E11. **(B)** Brightfield images of control **(a–e)** and experimental embryos **(f–j)**, showing eyelid **(a,f)**, fore- and hindlimb digit **(b,c,g,h)**, and lung **(d,e,i,j)** defects. Scale bar: **(a,f)** 2 mm; **(b,c,g,h)** 5 mm; **(d)** 750 μm; **(i)** 375 μm; **(e,j)** 25 μm. **(C)** Side-by-side comparison of control **(left)** and experimental lung.

Next, we examined the impact of Fgf10 inhibition during the E11–E12 time period (Figure 3A). Macroscopic examination of the craniofacial structures indicated impaired eye lid formation leading to an open eye phenotype in experimental vs. control embryos (compare eyelid in Figures 3Bf,a). This phenotype was previously described in *Fgf10* null embryos and caused by defective proliferation and coordinated migration of the epithelial leading edge cells to form the eyelid (Sekine et al., 1999; Tao, 2020). The analysis of the limb phenotype indicated that both forelimbs and hindlimbs formed in the experimental embryos (Figures 3Bg,h). However, close-up examination showed that the autopods (carpal/tarsal, metacarpal/metatarsal, phalanges) were truncated in both the forelimbs and hindlimbs (Figures 3Bg,h vs. b,c), a phenotype which was previously described upon inhibition of Fgfr2b ligands at E11.5 (Danopoulos et al., 2013). Finally, lung development was severely impaired in experimental embryos indicating that the impact of Fgf10 inhibition between E11 and E12 was no longer reversible (Figures 3Bd vs. Bi). Interestingly, the trachea and primary bronchi were clearly developed in experimental embryos. This is in sharp contrast with the lung lobes which were severely underdeveloped and did not branch properly (compare Figures 3Bj,e). We compared, immediately after birth, the experimental embryos arising from Dox-IP at E9.5 and E10.5 for their capacity to sustain normal breathing. Our results indicate that the E9.5 Dox-IP experimental embryos, while limbless, are viable, showing classical pink coloration and displaying normal respiratory movements. By contrast, E10.5 Dox-IP experimental embryos, while displaying significant limb formation, are all cyanotic and not moving, indicating lethality (see Supplementary Movie S1).

### Analysis of the Impact of Recombinant FGF10 on Early Human Alveolar Epithelial Progenitors Derived From iPSC or ESC Indicates Limited Overlap With the Previously Described “Fgf10 Transcriptomic Signature” Defined in Mouse Lung at E12.5

Human pluripotent stem cells have been used to study human lung development and disease. Complex protocols allowing directed differentiation of these cells toward the alveolar lineage have been published. These protocols involve precise temporal activation or repression of developmental pathways via the treatment *in vitro* of progenitor cells and their progeny by corresponding inhibitors and activators.

In the lung, FGF, WNT, BMP, SHH signaling pathways have been proposed to play remarkable roles in establishing the proximal-distal axis during human lung development (Hawkins and Kotton, 2015). Interestingly, most of these information are derived from mouse studies and the *in vitro* differentiation protocols are, at the best, mimicking what is known about the normal differentiation trajectory of the epithelial stem cells in the lung to give rise both to the alveolar and bronchiolar lineage. Given its important function during lung development, FGF10 is an integral part of the cocktail of growth factors used to progressively differentiate human stem cells toward the AT2 lineage (Huang et al., 2014).

**FIGURE 4:**
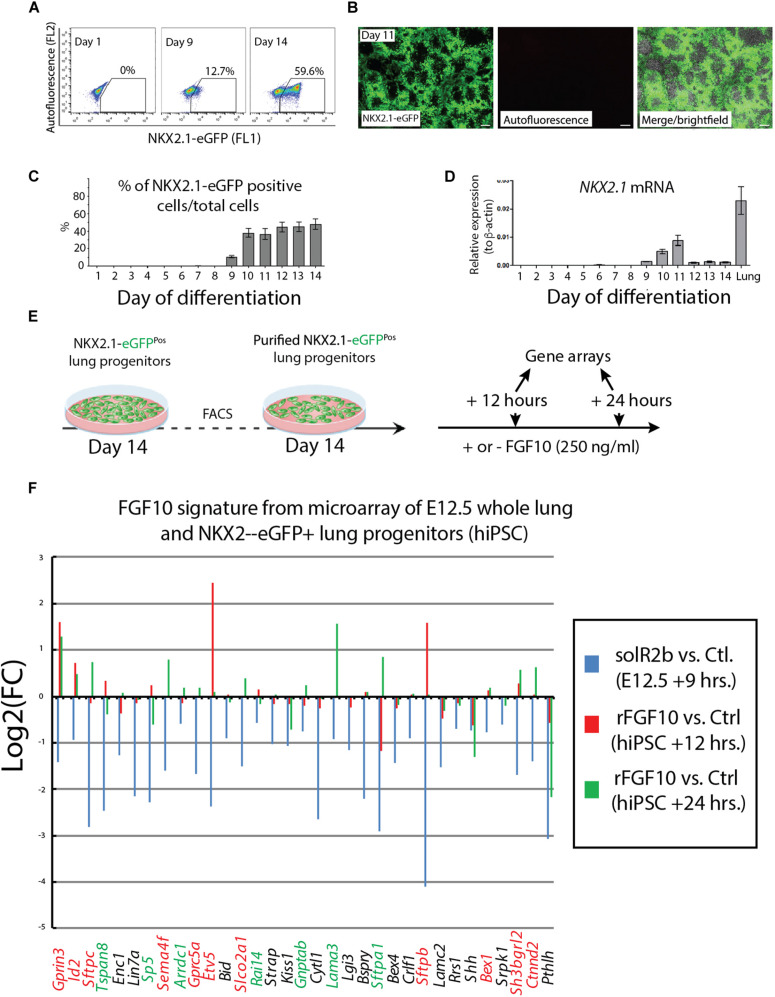
Directed differentiation culture of hiPSC to obtain purified NKX2.1-eGFP^+^ cells. **(A)** Fluorescence-activating analysis of cell culture of human cell line at different time points. **(B)** Quantification analysis of flow cytometry analysis. **(C)** Immunofluorescence microscopy analysis of GFP expression in cultured human cell line. **(D)** Quantification of NKX2.1 expression in human iPS cell line at different time points. **(E)** Human iPS cell line culture to get pure population of NKX2.1-eGFP^+^. **(F)** Microarray analysis of Fgf10 signature in control group (sFgfr2b) compared to human samples (hiPSC). Color code genes indicating different classes of Fgf10 targets.

NKX2.1 is the earliest known marker in lung cell fate specification (Hawkins et al., 2017). In this study, two human stem cell lines were used. Both the human embryonic stem cell (hESC) line (hES3_NKX2.1-eGFP) and the human induced pluripotent stem cell (hiPSC) line (MHHi006-A-2) were knock in lines for EGFP in the NKX2.1 locus and allowed, during the differentiation protocol, to use GFP expression as a surrogate to visualize and isolate by flow cytometry the NKX2.1-positive alveolar epithelial cells originating from the human stem cells. Cells were harvested at different time points from day 1 to day 14 and analyzed for GFP expression by using fluorescence-activated cell sorting (FACS) (Figure 4A) as well as qPCR. At day 9, 10% of the cells analyzed were positive for eGFP. This percentile increases to 60% within the 10–14 days culture period (Figure 4B). Direct visualization of GFP by immunofluorescence confirmed the flow cytometry data (Figure 4C). qPCR analysis indicated a relatively stable expression of *NKX2.1* during this time period (Figure 4D). For practical reasons regarding the availability of a sufficient number of human cells for our study, we chose day 14 as the time of isolation of the NKX2.1-eGFP alveolar progenitors. We then re-plated these cells and cultured them in presence or absence of 250 ng/ml of recombinant FGF10. We analyzed these cells by gene arrays after 12 and 24 h of treatment. Three samples for each human cell line for 12 h culture and 2 samples for each human cell line for 24 h culture (Figure 4E) were obtained. These samples, for each time point and condition, were pooled for the transcriptomic analysis.

**FIGURE 5:**
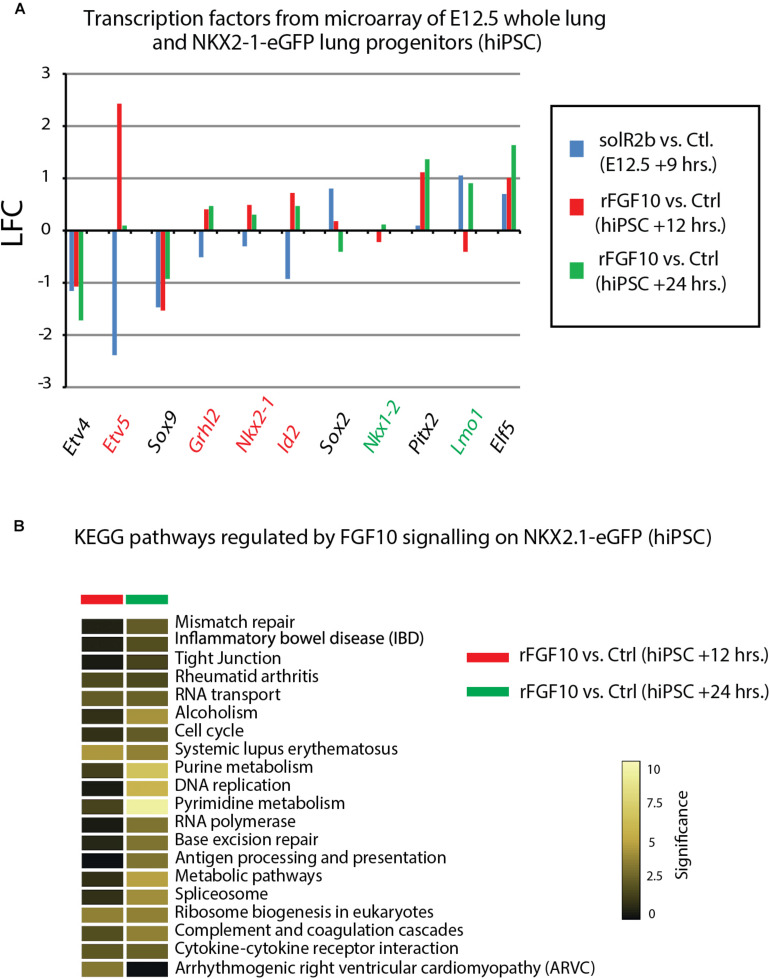
Further analysis of Fgf10 signaling targets. **(A)** Transcription factors which are Fgf10 direct targets. LFC was calculated so that it compares Fgf10 negative vs. Fgf10 positive expression. Therefore, the direction of each bar represents the effects of losing Fgf10 signaling. **(B)** KEGG pathways regulated by Fgf10 signaling.

Figure 4F uses, as a base for the analysis, the genes previously identified to be downstream of Fgf10 signaling at E12.5 in the mouse lung (Jones et al., 2019). This signature corresponds to a stage where in mouse, Fgf10’s role is mostly dedicated to the branching process. We analyzed the expression of these genes in the hiPSC treated vs. not treated with FGF10 for 12 h and 24 h. The rationale for this analysis is that the genes found to be part of the Fgf10 transcriptomic signature (which display downregulated expression in the comparison between soluble Fgfr2b and control lungs, see Jones et al., 2019 for details), should be upregulated in our gain of function experiment at least at one of the time points considered. Out of the 33 genes considered, only 11 were consistently conserved (these are written in red in Figure 4F and display upregulation at least at one of the times point eventually with no change at the other time point. Among them we found *ID2, SFTPC, SFTPB*, and *ETV5*) and seven partially conserved (these are written in green in Figure 4F and display upregulation of these genes at one of the time point with a downregulation at the other time point. Among them we found *SP5* and *SFTPA1*). Therefore, only 18 out of 33 genes (54%) of the genes identified as part of the mouse Fgf10 transcriptomic signature during the branching morphogenesis stage were conserved with alveolar epithelial progenitors derived from human iPSCs in our FGF10 gain of function experiment. A Similar result was obtained with hESC cells (Supplementary Figure S1). Interestingly, only five red genes (*ID2, SFTPC, SFTPB*, and *ETV5*) were overlapping between the two human cell lines. The significance of this result in terms of differentiation of alveolar epithelial cells obtained from the two human stem cell lines is not clear. We also examined the expression of transcription factors induced or repressed by Fgf10 in the mouse lung (Figure 5A). Out of the six genes induced by Fgf10 in mouse, four genes (67%) were also found to be upregulated in hiPSC upon treatment with FGF10. Among them, we found *ETV5*, *GRLH2*, *NKX2-1*, and *ID2*. By contrast only two out of the five transcription factors repressed by Fgf10 in the mouse lung, were also partially repressed in human iPSC, among them *NKX1-2* and *LMO1*. Similar results were obtained with hESC (Supplementary Figure S2A). Next, we examined the KEGG pathways regulated by FGF10 signaling in NKX2.1-eGFP hiPSC. We found mostly genes involved in DNA replication, Purine and Pyrimidine metabolism, Metabolic pathways (Figure 5B). At the exception of pathways involved in inflammation, similar results were obtained with the hESC (Supplementary Figure S2B).

## Discussion

**FIGURE 6:**
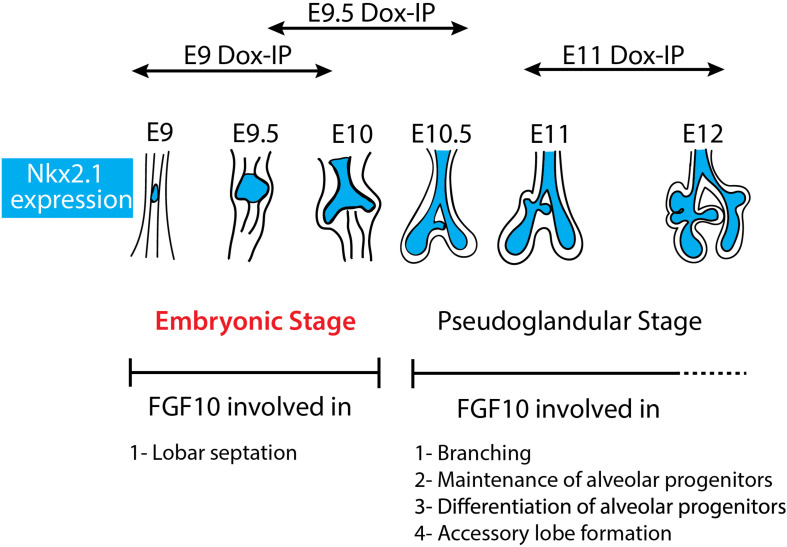
Schematic summary of Fgf10 signaling blockade at different time points. The impact of attenuating Fgfr2b ligand at different developmental time points (E9, E9.5, and E11) illustrates different function of Fgf10 on lung development and allowed to identify an early embryonic stage (in red) from E9 to E10. The main function of Fgf10 in the embryonic stage is in lobar septation leading to the four distinct lobes on the right lung. From E10.5, Fgf10 plays mostly a role in branching morphogenesis, alveolar progenitors maintenance and differentiation as well as in the formation of the accessory lobe which is the first lobe to appear on the right bronchus rudiment.

Our study indicates for the first time that during the E9–E12 time period, Fgf10 signaling plays several functions. One function is the control of lobar septation during the E9–E10 period (Figure 1). Lobar septation is the process by which the four lobes arising from the right lobe are separated from each other to form the cranial, medial, caudal, and accessory lobes. Interestingly, this function is not linked with the regulation of the branching process, which is not yet happening at this stage at the exception of the elongation of the two primary bud rudiments (see Figure 6).

The accessory lobe is differentially impacted at E9.0 Dox-IP (where this lobe is visible) and E9.5 Dox-IP (where it is not visible). Analysis of the lung branching pattern during early lung development indicates that the accessory lobe starts to form only after E10.5, thereby providing a reasonable explanation concerning the formation of a relatively normal accessory lobe following Fgf10 signaling inhibition from E9 to E10 (E9.0 Dox-IP). Interestingly, we previously reported that at E14.5, Fgf10 expression is found at the edges of the intralobular segments in the lung (Mailleux et al., 2005; El Agha et al., 2012) indicating that Fgf10 could play a role not only in subdividing the initial rudimentary right lobe in smaller lobes through the process of lobar septation but also the smaller lobes in lobules through a process of lobular septation. From E9.5 to E10.5 (E10 Dox-IP), our results indicate that Fgf10 is required for accessory (as well as cranial, medial, caudal) lobar septation as well as lobe growth. However, the impact of Fgf10 inhibition during this time period appears reversible as the lungs are functional at birth and display normal epithelial differentiation. By contrast, inhibition from E11 to E12 (E11 Dox-IP), leads to a complete arrest in the development of the lung leading to lethality at birth. The observation that the impact of Fgf10 inhibition is reversible at E9 Dox-IP and E9.5 Dox-IP but no longer at E11 Dox-IP supports the existence of different roles for Fgf10 signaling during this early time period of lung development. The regional expression pattern of *Fgf10* in the early lung may also explain the observed septation phenotype. We previously reported *Fgf10* expression using *in situ* hybridization in the early mouse lung (E9.75–E11.5) and found that *Fgf10* is expressed in the mesenchyme in the distal tip at E9.75 and appears to be more expressed in the right bronchi than in the left bronchi. This appears to be the case up to E12.5 (Bellusci et al., 1997). Regional expression of Fgf10 may therefore explain the lobular septation phenotype affecting the right lung.

This study allows now to possibly reconcile our previously published data concerning the absence of effect of FGF10 on the branching of human fetal lungs while mouse lungs at E12.5 responded by increased branch formation serving therefore as a positive control for the experiment (Danopoulos et al., 2019). Our results also indicate that it is potentially inaccurate to compare human fetal lungs at 12 weeks of gestation versus mouse lungs at E12.5 as they could correspond to two distinct developmental phases of lung development (embryonic phase in human versus pseudoglandular phase in mouse). As we now demonstrate that an embryonic phase, where Fgf10 signaling does not play a function, exists in mouse, it could be more relevant to compare the impact of FGF10 signaling in 12 weeks-old fetal human lungs with a mouse lung at the embryonic stage (E9–E10). One other important and relevant question concerns the duration of the pseudoglandular stage of human lung development, which is supposed to encompass week 4 through week 17 of gestation. Based on FGF10 activity as a read out, our results suggest that at 12 weeks of gestation, the human lungs are still in the equivalent of an “embryonic phase.” It is therefore still unclear when the pseudoglandular phase starts in humans. It is worth noting that mid-gestation in mouse is E9.5 where the lungs are not yet formed and the pups cannot possibly survive. In contrast, mid-gestation in humans is about 20 weeks of gestation, close to the limit of viability of severely premature babies.

More molecular and cellular insights into the different developmental phases and in particular the embryonic and pseudoglandular phases are clearly needed. In summary, our data presented herein indicate that the role of Fgf10 in the mouse and human lungs during development may not be so discordant after all, if we take into account the size scale and time difference in the development of mouse and human lungs.

## Materials and Methods

### Animal Models and Ethics Statements

Animal experiments were performed at Children’s Hospital Los Angeles under the research protocols (31–08 and 3111) approved by the Animal Research Committee and in strict accordance with the recommendations in the Guide for the Care and Use of Laboratory Animals of the National Institutes of Health. The approval identification for Children’s Hospital Los Angeles is AAALAC A3276-01. Harvesting organs and tissues from wild type and mutant mice following euthanasia using pentobarbital was approved at Justus Liebig University Giessen by the federal authorities for animal research of the Regierungspräsidium Giessen, Hessen, Germany (Approved Protocol GI 20/10 Nr. G 84/2016). All mice used to generate experimental and control embryos were housed in a specific pathogen free (SPF) environment with free access to food and water. Up to five females were housed together, while males were housed singly. Females between 9 and 12 weeks of age were used to generate embryos. *In vivo* mouse model to inhibit Fgfr2b ligands *in vivo* studies were conducted using an inducible dominant negative mouse model: *Rosa26^rtTA/+^; Tg(tet(o)sFgfr2b)*/+ (B6-Cg-Gt(ROSA)26Sortm1.1(rtTA,EGFP)Nagy Tg(tetO-Fgfr2b/lgh)1.3Jaw/sbel). This mouse model employs a reverse tetracycline transactivator (rtTA) under the transcriptional control of the ubiquitous Rosa26 locus. Upon administration of doxycycline, the rtTA is able to bind to the tetracycline operator (tetO), inducing the transcription of a soluble dominant negative form of Fgfr2b (sFgfr2b). These mice were generated by crossing *Rosa26^rtTA/+^* (Gt(ROSA)26SorTm1.1(rtTA,EGFP)Nagy) with *Tg(tet(o)sFgfr2b)/+* mice (Tg(tetO-Fgfr2b/Igh)1.3Jaw, obtained from Dr. Jeffrey Whitsett, Cincinnati, OH, United States). Experimental (*Rosa26^*rtTA/rtTA*^;Tg(tet(o)sFgfr2b*)/+) and littermate control (*Rosa26^*rtTA/rtTA*^; +/+*) embryos were generated by crossing *Rosa26^*rtTA/rtTA*^; Tg(tet(o)sFgfr2b)/+* and *Rosa26^*rtTA/rtTA*^; +/+* animals. Timed-pregnant females were used to conduct *in vivo* experiments. Doxycycline was administered at the desired embryonic time point (E) (E9, E9.5, and E11) via an intraperitoneal injection (i.p., Dosage: 0.0015 mg doxycycline/g mouse weight), where E0.5 was assumed to be noon on the day a vaginal copulation plug was found. At a determined time, post-doxycycline, i.p., a lethal dose of pentobarbital sodium was administered to animals via (i.p., Dosage: 0.4 mg pentobarbital/g mouse weight). After breathing ceased and a lack of pupil response to light was observed, cervical dislocation was performed to ensure death. Embryos were then harvested and washed in PBS with gentle shaking for 2 min. Embryonic lungs were then dissected and prepared for subsequent analyses.

### Immunofluorescence Staining

Lungs were collected at E18.5 then washed in sterile PBS (2 × 5 min), fixed in 4% PFA for 20 min on ice, and washed again (3 × 5 min). Lungs were dehydrated by successive washes in a graded ethanol series (30, 50, 70, and 100%) for 5 min each. To embed the lungs in paraffin, they were processed through the standard protocol. Before antibody staining, sections were first washed with gentle shaking in Xylol (3 × 10 min), and then in serial dilutions of ethanol (100, 70, 50, and 30%) for 3 min each, and finally in distilled water for 5 min. For each staining, an antigen retrieval step was performed, which involved incubating the slides in 75–90°C citrate-based antigen unmasking solution (pH 6.0; Vector Laboratories, Peterborough, United Kingdom) for 15 min and then cooling on ice for 30 min. Sections were then washed with PBST (1x PBS + 0.1% TWEEN20; 3 × 5 min). Blocking solution (1x PBS + 3% BSA + 0.4% Triton X) was then added atop each section for 1 h at room temperature. Primary antibodies were added to incubation buffer (1x PBS + 1.5% BSA + 0.2% Triton X) and samples were incubated overnight at 4°C against for CDH1 (1:100) (Mouse monoclonal anti-CDH1-FITC BD Biosciences Cat. # 612130), Pro-SPC (1:250) (Anti-Pro surfactant Protein C, polyclonal Ab Cat. # AB3786), and SOX9 (1:200) (Rabbit polyclonal anti-SOX9 Novus Biologicals Cat. # NBP 1-85551) dilution.

Following primary antibody incubation, samples were washed in PBST (3 × 5 min) and secondary antibodies were added (all at a 1:500 dilution) for 1 h at room temperature, in the dark. Samples were washed in PBST (3 × 10 min) and PBS for 5 min, with gentle shaking. Finally, ProLong Gold Antifade reagent with DAPI (Invitrogen, Schwerte, Germany) was added to each section and covered with a glass coverslip. Sections were imaged on a Leica DM 5500B upright fluorescent microscope system, with a DFC 360FX camera, and Leica Application Suite Advanced Fluorescence imaging software. Signal intensity was optimized to either a control or experimental sample for an experiment, and the acquisition and intensity values were similarly applied to each sample in that experiment, thus ensuring valid comparisons.

### Quantitative RT-PCR

Left lobes of lungs for each experiment (E9, E9.5) were collected at E18.5 in 700 μl QIAzol Lysis Reagent (Qiagen, Hilden, Germany). To isolate total RNA, the samples were transferred to gentle MACS M Tubes and homogenized in a gentle MACS Dissociator (Miltenyi Biotec) for 1 min. Total RNA was then isolated using the miRNeasy Mini Kit (Qiagen, Hilden, Germany), and eluted in 30 μl RNase-free water. RNA amount and purity were assessed with a NanoDrop 2000c (Thermo Scientific). Up to 1 μg of total RNA for each sample was then reverse transcribed using the QuantiTect Reverse Transcription Kit (Qiagen, Hilden, Germany). Primers were designed to amplify specific mature mRNAs using NCBI’s primer-BLAST option. Primers were further validated by PCR-based gel electrophoresis. qPCR reaction mixtures were set up using the PowerUp SYBR Green Master Mix (Thermo Fisher, Schwerte, Germany), with a final volume of 20 μl for each reaction. Reaction mixtures included the following components: 10 μl of 2X PowerUp SYBR Green Master Mix, between 300 and 800 nM of each primer, between 1 and 10 ng cDNA, and nuclease-free water. Samples were run with two or three technical replicates on a LightCycler 480 II (Celli et al.) using the following protocol: UDG activation at 50°C for 2 min; DNA polymerase activation at 95°C for 2 min; and 40 cycles of denaturation at 95°C for 15 s, annealing at 60°C for 15 s, and extension at 72°C for 1 min. To validate amplification specificity, a dissociation step was also included for each sample. Threshold cycles (Ct) were calculated and used for relative expression analyses, using mouse *Hprt* as the reference gene.

### Directed Differentiation of Human Pluripotent Stem Cells to Respiratory Progenitor Cells

In this study, we used two human pluripotent stem cell (hESC) lines; a hESC reporter line (hES3_NKX2.1-eGFP) and hiPSC NKX2.1 reporter line (MHHi006-A-2). Undifferentiated human pluripotent stem cells were differentiated first to definitive endoderm (DE) and then for further differentiation toward anterior foregut endoderm (AFE). For these two steps, we used different optimized media between day (−2) and day 4. For the first 2 days Rock-inhibitor was used to increase the viability of stem progenitor cells. When cells reached high density, (2.2 × 10^5^ cells/cm^2^) were seeded in KnockOut DMEM supplemented with 5% Serum replacement, 1% L-glutamine, 1% Non-essential amino acids, 1% Penicillin/Streptomycin, 0.46 mM 1-Thioglycerol (basal medium) supplemented with 3 μM Dorsomorphin and 10 μM SB435142 for 2 days, followed by 2 days of basal medium supplemented with 2 μM IWP2 and 10 μM SB435142. In order to allow cells to differentiate into distal lung epithelial progenitors, we used Dorsomorphin to inhibit bone morphogenic proteins signaling (BMP), IWP2 to inhibit wingless/integrase 1 (Wnt), and SB435142 to inhibit TGFb signaling. From day 8 to day 14, AFE cells were further differentiated toward NKX2.1 expressing lung progenitor cells by incubation in basal medium supplemented with 10 ng/ml BMP-4, 3 μM CHIR99021 and 10 ng/ml FGF-10 with daily medium exchanges. At day 14, cells were harvested in order to sort enriched population of NKX2.1-eGFP^+^ cells. Sorted cells were reseeded and treated with FGF10 (250 ng/ml) for 12 and 24 h. After each time point, cells were harvested for RNA isolation. Isolated RNA from collected samples were subjected to gene array. Three samples per each cell lines were used for 12 h culture and two samples for 24 h culture. All details of all used materials in directed differentiated, FACS and qPCR protocols have been previously published (Goulburn et al., 2011; Olmer et al., 2019).

### Microarray Analysis of Differentiated Human Alveolar Progenitors Treated or Not With FGF10- Comparison With the Fgf10 Transcriptomic Signature Identified in the Mouse Lung at E12.5

Sorted cells (enriched NKX2.1-eGFP^+^) after 14 days culture from human cell lines were cultured *in vitro* for 12 or 24 h in presence of FGF10 or PBS. At 12 and 24 h, RNA was extracted from these cells and processed for gene array.

Depending on the amount of RNA isolated per sample in an experiment, one of two possible microarray protocols was used. For RNA concentrations >50 ng/μl, the T7-protocol was followed. In this protocol, purified total RNA was amplified and Cy3-labeled using the LIRAK kit (Agilent Technologies, Waldbronn, Germany) following the kit instructions. Per reaction, 200 ng of total RNA was used. The Cy3-labeled aRNA was hybridized overnight to 8 Å∼ 60 K 60mer oligonucleotide spotted microarray slides (Agilent Technologies, design ID028005). For experiments where samples yielded <50 ng/μl of RNA, the SPIA-protocol was utilized. In this protocol, purified total RNA was amplified using the Ovation PicoSL WTA System V2 kit (NuGEN Technologies, Leek, Netherlands). Per sample, 2 μg amplified cDNA was Cy-labeled using the SureTag DNA labeling kit (Agilent Technologies). The Cy3-labeled aRNA was hybridized overnight to 8 Å∼ 60 K 60mer oligonucleotide spotted microarray slides (Agilent Technologies, design ID 074809 for mouse sample and design ID 072363 for human sample). For each protocol, hybridization, and subsequent washing and drying of the slides were performed following the Agilent hybridization protocol. The dried slides were scanned at 2 μm/pixel resolution using the InnoScan is900 (Innopsys). Image analysis was performed with Mapix 6.5.0 software, and calculated values for all spots were saved as GenePix results files. Stored data were evaluated using the R software (version 3.3.2) and the limma package (version 3.30.13) from Bioconductor. Gene annotation was supplemented by NCBI gene IDs via biomaRt (last accessed for mouse sample 08–03–2018). The data from the microarray experiments have been deposited in the NCBI’s gene expression omnibus GEO accession GSE124157 for E12.5 mouse lung sample and GEO Submission (GSE152597) [NCBI tracking system #21036195] for human samples.

## Data Availability Statement

The raw data supporting the conclusions of this article will be made available by the authors, without undue reservation.

## Ethics Statement

The animal study was reviewed and approved by the Animal experiments were performed at Children’s Hospital Los Angeles under the research protocols (31–08 and 3111) approved by the Animal Research Committee and in strict accordance with the recommendations in the Guide for the Care and Use of Laboratory Animals of the National Institutes of Health. The approval identification for Children’s Hospital Los Angeles is AAALAC A3276-01. Harvesting organs and tissues from wild type and mutant mice following euthanasia using pentobarbital was approved at Justus Liebig University Giessen by the federal authorities for animal research of the Regierungspräsidium Giessen, Hessen, Germany (Approved Protocol GI 20/10 Nr. G 84/2016). Written informed consent was obtained from the owners for the participation of their animals in this study.

## Author Contributions

ST, MJ, SD, CGS, CC, and DA were responsible for the mouse experiments. RO, SU, and UM carried out the experiments with the human cells. JW carried out the bioinformatic analysis. ST, SB, CSC, and DA conceived the project and wrote the manuscript. All authors contributed to the article and approved the submitted version.

## Conflict of Interest

The authors declare that the research was conducted in the absence of any commercial or financial relationships that could be construed as a potential conflict of interest.

## Supplementary Material

Supplementary Figure 1 Directed differentiation culture of hESC to reach purified NKX2.1-eGFP^+^**(A)** Fluorescence-activating analysis of cell culture of human cell line at different time points. **(B)** Quantification analysis of flow cytometry analysis. **(C)** Immunofluorescence microscopy analysis of GFP expression in cultured human cell line. **(D)** Quantification of NKX2.1 expression in human ES cell line at different time points. **(E)** Human ES cell line culture to get pure population of NKX2.1-eGFP^+^. **(F)** Microarray analysis of Fgf10 signature in control group (sFgfr2b) compared to human samples (hESC). Color code genes indicating different classes of Fgf10 targets.

Supplementary Figure 2 Microarray analysis of hESC culture compared to control (sFgfr2b). **(A)**Transcription factors which are Fgf10 direct targets. LFC was calculated so that it compares Fgf10 negative vs. Fgf10 positive expression. Therefore, the direction of each bar represents the effects of losing Fgf10 signaling. **(B)** KEGG pathways regulated by Fgf10 signaling.

Supplementary Table 1 List of qPCR primer sequences.
