# Supplementary figures and images for: Fgf10 Signaling-Based Evidence for the Existence of an Embryonic Stage Distinct From the Pseudoglandular Stage During Mouse Lung Development
Source: Front Cell Dev Biol. 2020 Oct 22;8:576604. doi: 10.3389/fcell.2020.576604 (PMC7642470; doi:10.3389/fcell.2020.576604)

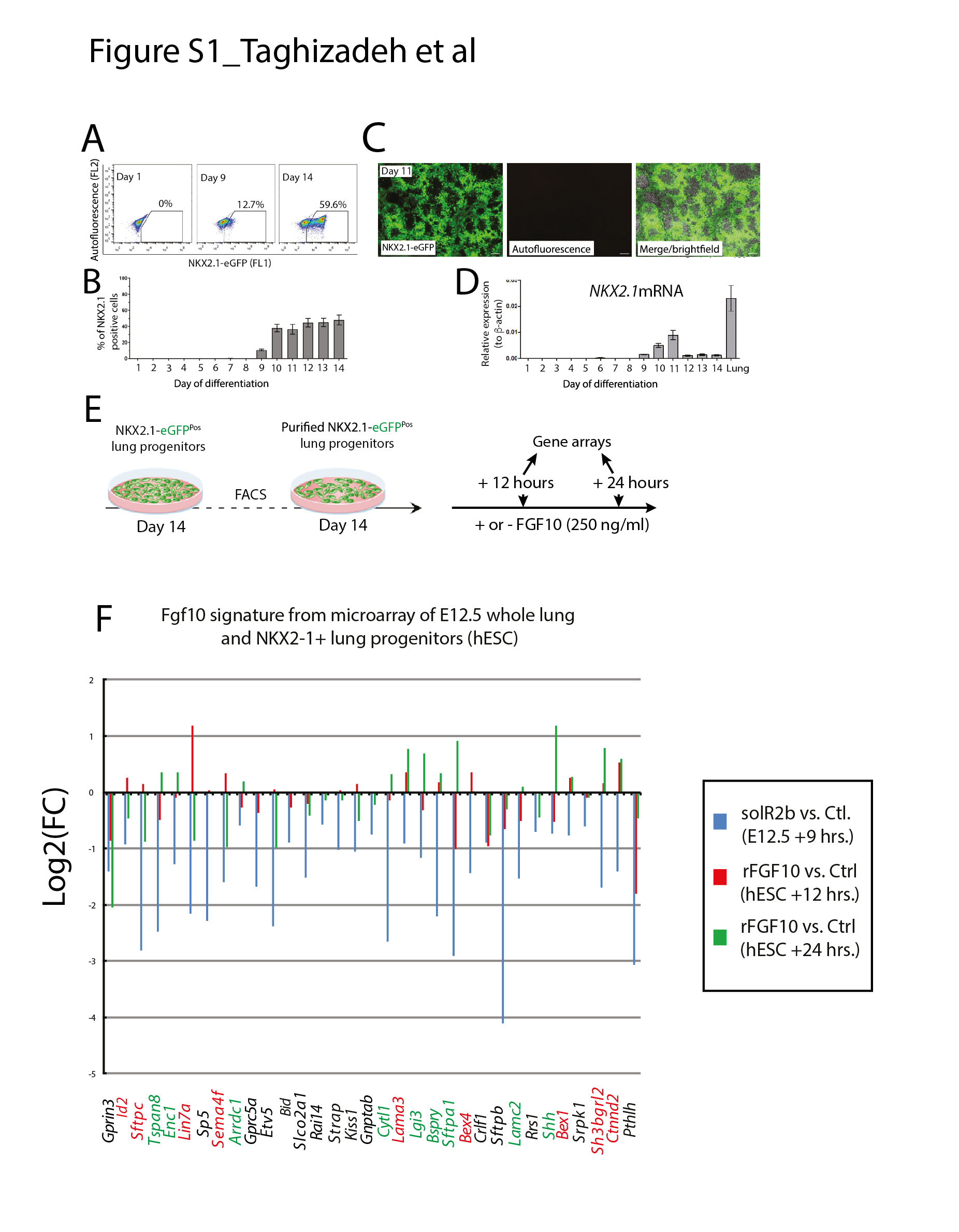

Supplement: Supplementary Figure 1 — Directed differentiation culture of hESC to reach purified NKX2.1-eGFP+(A) Fluorescence-activating analysis of cell culture of human cell line at different time points. (B) Quantification analysis of flow cytometry analysis. (C) Immunofluorescence microscopy analysis of GFP expression in cultured human cell line. (D) Quantification of NKX2.1 expression in human ES cell line at different time points. (E) Human ES cell line culture to get pure population of NKX2.1-eGFP+. (F) Microarray analysis of Fgf10 signature in control group (sFgfr2b) compared to human samples (hESC). Color code genes indicating different classes of Fgf10 targets. [file Image_1.JPEG]

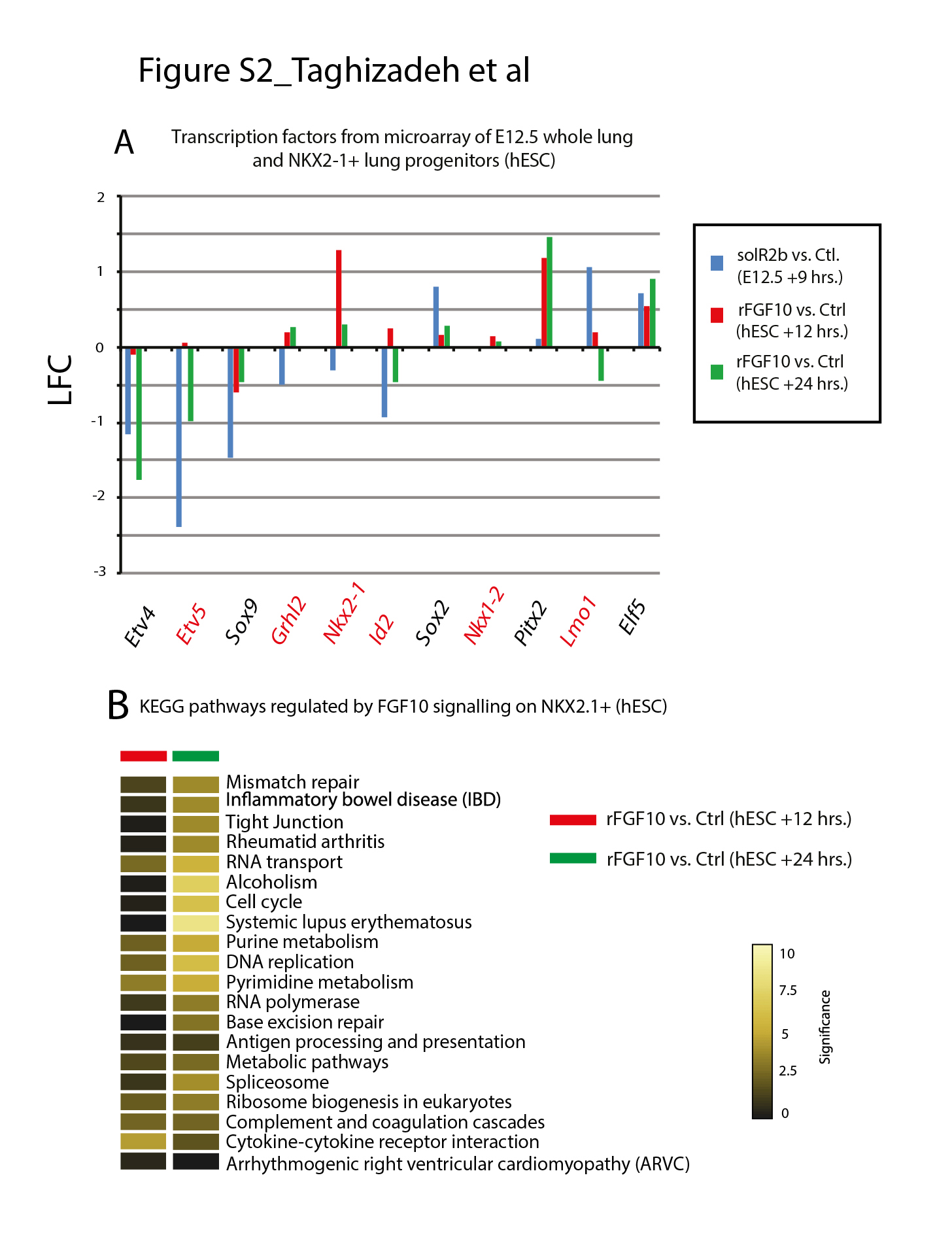

Supplement: Supplementary Figure 2 — Microarray analysis of hESC culture compared to control (sFgfr2b). (A)Transcription factors which are Fgf10 direct targets. LFC was calculated so that it compares Fgf10 negative vs. Fgf10 positive expression. Therefore, the direction of each bar represents the effects of losing Fgf10 signaling. (B) KEGG pathways regulated by Fgf10 signaling. [file Image_2.JPEG]
