# Supplementary material for: Fgf10 Signaling-Based Evidence for the Existence of an Embryonic Stage Distinct From the Pseudoglandular Stage During Mouse Lung Development
Source: Front Cell Dev Biol. 2020 Oct 22;8:576604. doi: 10.3389/fcell.2020.576604 (PMC7642470; doi:10.3389/fcell.2020.576604)
Supplement: Supplementary Table 1 — List of qPCR primer sequences. [file Table_1.pdf]

Supplementary Table S1 - List of qPCR primer sequences

Genes	Forward Primer	Reverse Primer
Etv4	AGGAGTACCATGACCCCCTG	GGACATCTGAGTCGTAGGCG
Etv5	TAGCTGAAGCACAAGTTCCTGA	GCAGCTCCCGTTTGATCTTG
Sox2	GGCAGAGAAGAGAGTGTTTGC	TCTTCTTTCTCCCAGCCCTA
Sox9	AGTCGGTGAAGAACGGACAA	CTGAGATTGCCCAGAGTGC
Sftpc	GGTCCTGATGGAGAGTCCAC	GATGAGAAGGCGTTTGAGGT
Aqp5	TAACCTGGCCGTCAATGC	GCCAGCTGGAAAGTCAAGAT
Hprt	TCCTCCTCAGACCGCTTTTT	ATCATCGCTAATCACGACGC
